# Internet-based treatment for Romanian adults with panic disorder: protocol of a randomized controlled trial comparing a Skype-guided with an unguided self-help intervention (the PAXPD study)

**DOI:** 10.1186/s12888-016-0709-9

**Published:** 2016-01-14

**Authors:** Amalia Maria Ciuca, Thomas Berger, Liviu George Crişan, Mircea Miclea

**Affiliations:** Department of Clinical Psychology and Psychotherapy, University of Bern, Bern, Switzerland; Department of Psychology, Babeş-Bolyai University, Cluj-Napoca, Romania

**Keywords:** Panic disorder, Randomized controlled trial (RCT), Internet-based cognitive-behavioral treatment (ICBT), Self-help, Guided self-help, Real-time communication, PAXonline

## Abstract

**Background:**

Efficacy of self-help internet-based cognitive behavior therapy (ICBT) for anxiety disorders has been confirmed in several randomized controlled trials. However, the amount and type of therapist guidance needed in ICBT are still under debate. Previous studies have shown divergent results regarding the role of therapist guidance and its impact on treatment outcome. This issue is central to the development of ICBT programs and needs to be addressed directly. The present study aims to compare the benefits of regular therapist guidance via online real-time audio-video communication (i.e. Skype) to no therapist guidance during a 12-week Romanian self-help ICBT program for Panic Disorder. Both treatments are compared to a waiting-list control group.

**Methods/Design:**

A parallel group randomized controlled trial is proposed. The participants, 192 Romanian adults fulfilling diagnostic criteria for panic disorder according to a diagnostic interview, conducted via secured Skype or telephone, are randomly assigned to one of the three conditions: independent use of the internet-based self-help program PAXonline, the same self-help treatment with regular therapist support via secured Skype, and waiting-list control group. The primary outcomes are severity of self-report panic symptoms (PDSS-SR) and diagnostic status (assessors are blind to group assignment), at the end of the intervention (12 weeks) and at follow-up (months 3 and 6). The secondary measures address symptoms of comorbid anxiety disorders, depression, quality of life, adherence and satisfaction with ICBT. Additional measures of socio-demographic characteristics, personality traits, treatment expectancies, catastrophic cognitions, body vigilance and working alliance are considered as potential moderators and/ or mediators of treatment outcome.

**Discussion:**

To the best of our knowledge, the present study is the first effort to investigate the efficacy of a self-help internet-based intervention with therapist guidance via real-time video communication. A direct comparison between therapist guided versus unguided self-directed intervention for panic disorder will also be addressed for the first time. Findings from this study will inform researchers and practitioners about the added value of online video-therapy guidance sessions and the type of patients who may benefit the most from guided and unguided ICBT for Panic disorder.

**Trial registration:**

ACTRN12614000547640 (Australian New Zealand Clinical Trials Registry). Registered 22/05/2014.

## Background

Hundreds of millions of people are affected by mental health disorders worldwide and failing to adequately address this issue brings significant suffering, disability and economic loss. The lifetime prevalence for mental health disorders in general is above 46 %, with the highest prevalence recorded for anxiety disorders, given that almost 1 out of 3 people (28.8 %) suffer from an anxiety disorder during their lifetime [1]. Every year over 38.2 % (164.7 million people) of the total EU population suffer from at least one mental disorder and the resulting economic costs rise at 3–4 % of EU Gross Domestic Product (GDP) [2].

One third of medical consultations made by general practitioners are in fact provided for mental health problems [3–5]. A report released by WHO (World Health Organization) and WONCA (World Organization of Family Doctors) in 2008 stated that “up to 60 % of people attending primary care clinics have a diagnosable mental disorder” (page VII) [6]. There is an alarming underdetection of mental health problems – for example, detection rates of anxiety and mood disorders in general practice reach only 15 and 36 %, respectively [7, 8]. Moreover, collaboration between GPs and mental health services is either poor or non-existent.

Despite the potential to successfully treat mental disorders, only a small minority receive even the most basic treatment. Studies have shown that 60 % of those in need receive no treatment at all, and only 32.7 % of those who receive treatment get it at minimal quality standards [9]. Furthermore, there are numerous individual and systemic barriers to accessing mental health services [10], thus the treatment is provided only with a significant delay from the onset of the disorder (after 9–23 years for anxiety disorders and 6–8 years for mood disorders) [11]. In some countries, the situation is even worse. In Romania, where the current study is conducted, 76.4 % of the individuals with mental health disorders do not have access to any form of treatment; only about 11.5 % receive psychological or psychiatric help [12].

Computer and Internet-based cognitive behavioral treatments (ICBT) represent recent attempts to innovate the psychotherapeutic process, matching both the existent demands of the market, and the new attitudes of those who seek help in the digital era. Research on internet-based treatments has grown rapidly in the past decade, gathering more than 100 controlled studies that tested various intervention programs for anxiety disorders, mood disorders, or other psychiatric conditions [13–15]. Overall, the outcomes of these studies have been positive and very convincing. Several studies reported no significant differences in treatment outcomes when comparing internet-based interventions to traditional face-to-face therapy [16–21]. For a review see Andersson et al. [22]. Because half of all lifetime cases of mental disorders start by age 14 years and the median age of onset for anxiety is 11 years [1], it should be mentioned that ICBT shows promising results not just for adults, but also for treating anxiety and depression in youth [23, 24]. Anxiety disorders seem to be one of the most approachable and treatable types of mental disorder through online therapy [25–31]. Panic disorder (PD) with or without agoraphobia is one of the conditions for which ICBT programs have been developed since the late 1990s and there are several empirical studies supporting their efficacy and effectiveness [13, 17, 19, 32–42]. In Romania, only one internet-based treatment has been tested so far, the Swedish program Sofie [43]. The study yielded positive outcomes for the treatment of social anxiety disorder, but the program is currently not in use.

The most promising interventions designed and tested so far are guided self-help interventions. A guided ICBT intervention is a web-based self-help program combined with minimal, but regular therapist support. In guided programs, asynchronous communication (emails or text messages) is most commonly used and, on average, patient guidance does not take more than 10 min per week [44]. So far, research is very limited regarding web-based treatment programs that provide guidance through synchronous (real time) audio-video communication (e.g. video chat programs such as Skype). On the other hand, there is growing evidence that the efficacy of CBT delivered via videoconference is equivalent to face-to-face treatment for mood and anxiety disorders [45–50]. This type of communication closely matches face-to-face therapy and enables access to important face-to-face cues such as verbal tone, facial expressions, and body language. Through synchronous guidance, the therapists may be able to offer more individually tailored feedback and support according to each patient’s personal needs. Furthermore, this type of contact may facilitate higher engagement, feelings of accountability and social support, and reduce the risk of misunderstandings, compared to emails and instant messaging [51]. All of the above could also facilitate the development of a better therapeutic alliance [52], a factor that has consistently proven to be a key predictor for therapeutic change in face-to-face therapy [53, 54]. So far, there is mixed evidence regarding the importance of the therapeutic alliance in guided ICBT [44]. Even if contact with the therapist is only minimal, patients seem to develop a strong alliance, but this makes no difference for the outcome, at least for self-help interventions with rather low-intensity guidance [44]. However, the therapeutic alliance in internet-based treatments that include video conferencing is yet to be investigated [55]. Of course, there are also disadvantages for using real-time audio-video communication. For instance, compared to asynchronous written exchanges, patients do not have the opportunity to revisit and reread the dialogues with their therapists [51]. The patients are still able to revisit and reflect upon the information enclosed in the treatment modules, but they cannot review their guidance sessions.

It is still not clear enough whether guided online treatments work better than unguided treatments [18, 31, 56–59], however there is a strong tendency for the superiority of guided treatment, especially for depression [14, 60]. So far, the literature suggests a superiority of guided versus unguided self-help treatments in terms of efficacy, adherence to treatment and drop-out rates [14, 61–63], but the conclusions are based on few studies and mainly limited to depression and social anxiety disorder, thus restricting the generalizability of the findings [64].

There is good quality evidence that ICBT treatments, especially guided interventions, are both efficacious and effective, either compared with waiting-list or with face to face interventions, but there is no intervention that is suitable for everyone [44]. In order to be able to make recommendations for a certain person, with certain characteristics, we need to know which variables can predict or mediate/ moderate the outcomes. Unfortunately, the studies conducted so far showed that there are few stable predictors across studies and diagnoses [65]. For example, treatment adherence was considered as a potential predictor variable, but there are inconsistencies, with some studies finding an association between adherence and clinical outcomes [66–68] and others not [65, 69, 70]. Another example is the severity of the disorder at baseline. In one study, a high baseline severity predicted better outcomes [71], while another study showed the contrary, low levels of panic symptom severity predicting better outcomes [65]. Another interesting result concerns the role of comorbidity. A recent study showed that having a comorbid anxiety disorder was associated with a better treatment outcome among patients with panic disorder but not patients with social anxiety disorder [72], while others found no significant association [65, 73]. Considering these mixed results, we agree with Andersson who concluded “We do not know much about predictors of treatment outcome and the mechanisms involved in generating good outcome. Thus, more studies on moderators and mediators are needed” ([44], p.106).

### Objectives and research questions

The aim of the present trial is to investigate the clinical efficacy of an internet-based self-help treatment program for panic disorder with therapist guidance via secured Skype in Romanian adults. The program (PAXonline for panic disorder) has been developed de novo by the authors, based on empirically validated cognitive-behavioral models of anxiety disorders [74–76], and it is currently the only available psychological treatment of this kind in Romania. A secondary objective is to compare the guided self-help intervention to a similar but unguided intervention program, considering clinical outcomes, adherence to treatment and drop-out. Additional exploratory research will be conducted to identify potential predictors, moderators and mediators of treatment outcome, such as patients’ expectations, patient-therapist working alliance, panic disorders severity and comorbidity status, and other relevant factors that were chosen based on previous research findings.

## Methods/ Design

The study design is reported in line with the CONSORT 2010 statement (Consolidated Standards of Reporting Trials) [77] and the SPIRIT 2013 Statement (Standard Protocol Items: Recommendations for Interventional Trials) [78].

### Study design

This study is a randomized controlled trial with two active treatment conditions and a waiting list control group. A one-factor experimental design (parallel-group, superiority trial) will be used. The three conditions are:

1. *Web-based self-help program for panic disorder with intensive guidance (PAX-guided)*: participants in this group will complete the PAXonline panic disorder treatment program with support from a therapist, which include weekly emails and regular 15-30 min Skype sessions. In total, there will be 10 Skype sessions available.
2. *Web-based self-help program (PAX-unguided)*: participants in this group will complete the PAXonline panic disorder program in a self-guided format, i.e. without any support from a therapist.
3. *Waiting list control group*. This group will not receive any treatment for 12 weeks. At the end of the waiting period, the participants receive any of the two treatment programs, depending on their choice.

### Ethical approval

The study has been approved by the Ethical Review Board of The Center for the Management of Scientific Research Babes-Bolyai University (No. 31697/ 12.05.2014), and has been prospectively registered in Australian New Zealand Clinical Trials Registry (ACTRN12614000547640, 22/05/2014). See Table 1 for the WHO Trial Registration Data Set.

**Table 1:** Trial registration data set as recommend by World Health Organization (WHO)

Data category	Information
Primary registry and trial identifying number	Australian New Zealand Clinical Trial Registry (ANZCTR), ACTRN12614000547640
Date of registration in primary registry	22.05.2014
Secondary identifying numbers	Nil, U1111-1156-7294, 366360, 31697
Source(s) of monetary or material support	Babes-Bolyai University, Faculty of Psychology; Sciex - Scientific Exchange Programme NMS.CH; The Romanian Association for Online Counselling and Psychotherapy (ACPOR)
Primary sponsor	Babes-Bolyai University, Faculty of Psychology; 37, Republicii Street, Cluj-Napoca, 400015, Cluj
Secondary sponsor(s)	Sciex - Scientific Exchange Programme NMS.CH; CRUS Sciex-MPC, P.O. Box 607, CH-3000 Berne 9
	ACPOR- Cluj-Napoca, Romania
Contact for public queries	Babes-Bolyai University, School of Psychology and Educational Sciences, Department of Psychology
	Mr Mircea Miclea, Mr Liviu G. Crisan (mirceamiclea@gmail.com; liviugcrisan.neuro@gmail.com)
Contact for scientific queries	Ms Amalia Ciuca (amalia.ciuca@psy.unibe.ch; amaliaciuca@psychology.ro) Babes-Bolyai University, School of Psychology and Educational Sciences;
Public title	PAXonline: A Randomized Controlled Trial Assessing the Efficacy of an Internet-Based Cognitive Behavior Intervention for Panic Disorder
Scientific title	PAXonline: A Randomized Controlled Trial Comparing the Efficacy of an Internet-Based Cognitive Behavior Intervention, delivered with or without assistance from a therapist, to waiting-list in Romanian adults with Panic Disorder
Countries of recruitment	Romania, Spain, Italy
Health condition(s) or problem(s) studied	Mental health, anxiety disorders
Intervention(s)	Active comparators: Internet cognitive-behavioral treatment for panic disorder offered with or without assistance from a psychotherapist
	Placebo comparator: waiting list group.
Key inclusion and exclusion criteria	Inclusion criteria: diagnostic of Panic Disorder (confirmed by an experienced clinician through semi-structured clinical interview); age within the range of 18–65 years; access to a computer with internet connection; written informed consent provided; no participation in psychological treatment for panic disorder in the last 3 months.
	Exclusion criteria: severe comorbidities (e.g. bipolar disorders, psychotic disorders, substance abuse); mental retardation; suicidal ideation or behaviors; benzodiazepines treatment
Study type	Interventional
	Allocation: randomized; Intervention model: parallel assignment Masking: blind outcomes assessment
	Primary purpose: treatment
Date of first enrolment	28 May 2014
Target sample size	120 (the target sample size was changed to 192, but an official change was not made in the registry)
Recruitment status	Recruiting
Primary outcome(s)	Symptoms and severity of panic disorder (PDSS-SR); The Agoraphobic Cognitions Questionnaire; The Body Sensations Questionnaire
Key secondary outcomes	Symptoms and severity of depression - Patient Health Questionnaire-9 (PHQ-9); The Work and Social Adjustment Scale; the Working Alliance Inventory Short Revised (WAI-SR); Psychiatric Diagnostic and Screening Questionnaire (PDSQ); Panic Attack Cognition Questionnaire (PACQ); SS-5 - a 5-item shortened version of the Medical Outcomes Study Social Support Scale (MOS-SSS); Credibility/ Expectancy Questionnaire (CEQ); Body vigilance scale (BVS); Dependent personality disorder traits (Scale from OMNI-IV - Personality Disorder Inventory); System Usability Scale (SUS).

Issue date: 17.11.2015

Protocol amendment number: 02

Revision chronology

UM 00, 22.05.2014 Original

UM 01, 17.07.2015 Amendment 01: Changes were registered in Section 4 regarding secondary outcomes (OMNI-IV Dependent Personality Subscale was added) and in Section 8 regarding funding and sponsors (Sciex - Scientific Exchange Programme NMS.CH was added)

UM 02, 17.11.2015 Amendment 02: The time period for recruiting was extended

### Participants

Native Romanian speakers with Panic Disorder (*n* = 192) will be recruited via media (TV news, internet advertisements, and social media), promotional advertising displayed in emergency rooms, and direct recommendations made by a network of general practitioners and psychotherapists.

### Inclusion and exclusion criteria

Participants are included in the study if the following conditions are met: (1) their symptomatology fulfills the diagnostic criteria for panic disorder according to DSM-IV-TR (APA, 2000); (2) their age is within the range of 18–65 years; (3) they have access to a computer with internet connection; (4) they are native Romanian speakers; (5) they provide a written informed consent.

Participants are excluded if any of the following criteria is met: (1) they are currently enrolled in a different psychotherapeutic program or have received psychotherapy in the previous 3 months; (2) they present severe comorbidities, other than a different anxiety disorder or mild depression (e.g. bipolar disorders, psychotic disorders, substance abuse); (3) they suffer from mental retardation; (4) their symptoms are aggravated by a severe medical problem (e.g. ventricular tachycardia, heart attack, stroke, pulmonary fibrosis, hyperthyroidism, epilepsy); (5) they present suicidal ideation or behaviors.

Medication use is permitted, but only if the dosage was constant in the previous month and the same dosage must remain constant during the trial. Benzodiazepines are not allowed, according to existing recommendations [79–81]. All eligible participants must fill out and return a detailed informed consent form before starting the trial.

### Procedure

Individuals who struggle with panic attacks can freely access the study website which is publicly available at: http://studiu.paxonline.ro. The website provides information about panic disorder and treatment options, a description of the present clinical trial, and information about enrollment. The screening process for eligible participants is described below.

The participants start the screening process on the study website by filling out several online forms, anonymously. Firstly, participants must provide an online informed consent, then provide age and gender information. Next, they fill out three key questionnaires: (1) a screening for the exclusion criteria; (2) a scale for panic disorder diagnosis and severity (PDSS-SR); and (3) a screening for psychiatric comorbidities (PDSQ). Only participants who do not meet any exclusion criteria, who have a PDSS-SR score of at least 6 (see [82] for PDSS-SR cutoffs), and have completed the entire PDSQ screening are invited to provide an email address for further contact. Eligible participants are contacted by email to schedule a semi-structured clinical interview via secured Skype or telephone. The interviews are conducted by a trained clinical psychologist, who seeks to verify and confirm the diagnosis of a panic disorder and the compliance with all the inclusion/ exclusion criteria. At this point, participants who remain eligible after the interview receive an email from the principal investigator with a detailed informed consent and a link to fill out the online pre-treatment questionnaires. Participants are required to give their express consent to be included in the study. Specifically, they reply to the contact email confirming that they have read the informed consent and they accept to voluntarily participate in the study. They must also fill out their name and contact details at the end of the document. Participants who consent to this procedure are then randomly allocated to one of the three study groups.

After randomization, each participant receives an email containing instructions for the next phase. The guided participants receive a document with the recommended pathway for the entire course of treatment, along with instructions on how to communicate with their psychotherapist in the platform. They have a few days to explore the platform and read the first recommended module before scheduling their first Skype session with their psychotherapist. The first session is an introductory session in which the therapist explains the platform’s architecture and further collaboration between therapist and participant. All the Skype sessions will be audio recorded by the psychotherapists. To ensure treatment integrity and adherence to protocol, an impartial licensed clinical psychologist will randomly check 3 sessions from the work done with each patient.

We are using a secured Skype application for the guidance sessions. Skype is using a Peer-to-peer (“P2P”) technology, meaning that individuals communicate directly through their computers rather than through a third party host. All Skype-to-Skype voice, video, file transfers and instant messages are encrypted. Skype uses the AES (Advanced Encryption Standard), which is used by the US Government to protect sensitive information, and Skype also uses the strong 256-bit encryption. User public keys are certified by the Skype server at login using 1536 or 2048-bit RSA certificates [83].

The unguided participants receive a document with the recommended pathway for the entire course of treatment and are informed that they may ask for help with technical problems. This means they may ask for help if they are unable to access the platform, have problems with seeing movies, fill in documents or download resources. Although they receive strong recommendations to work through the modules in a certain order, there are no restrictions imposed and patients have access to all modules and resources, from the very beginning.

Both groups are asked to complete the recommended modules according to the instructions, and to fill out the PDSS-SR every two weeks. A pop-up reminder appears every time the participants access the platform if more than two weeks have passed since they last completed the questionnaire. During the treatment period, the participants receive a secured link by email, asking them to fill out the other self-report measurements at the established time points according to Table 2.

**Table 2:** Measurements and time of assessment

Instruments	Abbreviation	Aim	Time of assessment
Clinician administered			
Semi-structured clinical interview for DSM-IV axis I disorders	PDSQ	DSM-IV Axis I disorders	Pre and post intervention (12 weeks)
Self-report ratings			
Primary outcome measure			
Panic disorder severity scale - self report	PDSS-SR	Severity of panic symptoms	Pre-treatment, every two weeks after intervention commencement, mid-treatment, post-treatment, and at follow-up: months 1, 3, 6 and 12
Secondary outcome measures			
The agoraphobic cognitions questionnaire	ACQ	Maladaptive cognitions	Pre-treatment, mid-treatment, post-treatment, and at follow-up: months 1, 3, 6 and 12
The body sensations questionnaire	BSQ	Fear of body sensations	Pre-treatment, mid-treatment, post-treatment, and at follow-up: months 1, 3, 6 and 12
Patient health questionnaire-9	PHQ-9	Symptoms and severity of depression	Pre-treatment, mid-treatment, post-treatment, and at follow-up: months 1, 3, 6 and 12
The work and social adjustment scale	WSAS	Functional impairment	Pre-treatment, mid-treatment, post-treatment, and at follow-up: months 1, 3, 6 and 12
Psychiatric diagnostic and screening questionnaire	PDSQ	Axis 1 disorders	Pre-treatment, post-treatment, and at follow-up: months 6 and 12
Panic attack cognition questionnaire	PACQ	Catastrophic cognitions	Pre-treatment, mid-treatment, post-treatment, and at follow-ups
Body vigilance scale	BVS	Attentional focus on body sensations	Pre-treatment, mid-treatment, post-treatment, and at follow-ups
Perceived stress scale – 10	PSS-10	Perceived stress levels	Pre-treatment, mid-treatment, post-treatment, and at follow-ups
Additional measures			
Working alliance inventory short form	WAI-S	Therapeutic alliance	Administered after first and fourth session
Credibility/ expectancy questionnaire	CEQ	Expectancy for change and treatment credibility	Administered at 2 and 6 weeks during intervention
System usability scale	SUS	Usability and learnability	Administered at 2 and 6 weeks during intervention
a 5-item shortened version of the Medical Outcomes Study Social Support Scale (MOS-SSS)	SS-5	Social support	Pre-treatment, mid-treatment, post-treatment, and at follow-ups
OMNI-IV personality disorder inventory	OMNI-IV	Dependent personality	Pre-treatment
Personal autonomy questionnaire	PAQ	Autonomy	Pre-treatment
Psycho-education questionnaire	PEQ-8	Panic disorder knowledge	Pre and post-treatment
Drop-out reasons questionnaire	DRQ-18	Drop-out reasons	After dropping out
Patient feedback questionnaire	PFQ	Patient satisfaction	Post-treatment

The waiting list control group does not receive treatment for 12 weeks. Throughout this waiting period, the participants in this group are required to fill out all the scheduled questionnaires - at pre-randomization, after 6 weeks, and at the end of the 12 weeks period. Afterwards, they can choose to follow the PAXonline treatment for panic disorder either independently or with guidance from a therapist. For an overview of measurements and time of assessment please see Table 2. After the last follow-up evaluation, additional treatment (4 weeks using the intervention program and one Skype-session) is available upon request for all participants in the study.

### Interventions

The PAXonline Program for Panic Disorder is a 12-week internet-based treatment, which consists of cognitive-behavioral therapy modules, delivered with or without assistance from a therapist. The Panic Disorder Program contains 16 modules which address important cognitive behavioral psychotherapy elements such as: psychoeducation on the disorder and means of intervention; techniques for decreasing neurophysiological hyperarousal; cognitive restructuring; exposure to feared somatic sensations, alongside with situational (in vivo) exposures to reduce agoraphobic avoidance; positive emotions training; problem-solving training; behavioral activation and cognitive restructuring exercises to reduce symptoms of depression; relapse prevention. The treatment modules and the manual used to guide the intervention in our RCT were written based on empirically validated cognitive-behavioral models of anxiety disorders [74–76]. Each module can be completed in 15–40 min and the participants are provided with a recommended timetable (one or two modules per week, depending on the complexity of the content and the homework assignments). For a more detailed description of the intervention modules please refer to Table 3.

**Table 3:** Paxonline panic disorder treatment

Modules	Short description
Introductory module. *Anxiety, from normality to pathology*	Psychoeducation concerning fear, anxiety, fight or flight response, the brain circuits involved in the fear response, etiology of anxiety and treatment directions. Normalizes patients’ reactions and sets correct expectancies.
Module 1. *Understand what’s happening with you*	Introductory information on panic attacks, panic symptoms and how panic disorder develops. Study cases and video illustrations. Patients begin to register PD related behaviors and thoughts using online worksheets.
Module 2. *Understand what you have to do*	Introducing treatment strategies, the treatment plan and boosting confidence and motivation for change.
Module 3. *Reduce hyperactivation through breathing retraining*	Information on the effects of hyperventilation, breathing regulation and abdominal breathing exercises.
Modules 4 and 5. *Reduce hyperactivation through autogenic training*	Inducing relaxation by using autogenic training exercises. Videos and audio files are provided for practice.
Module 6. *Reduce hyperactivation through physical exercises*	Psychological and biological benefits of physical exercises are presented; how to use them regularly.
Module 7. *Optimizing attentional functioning*	Attention biases in anxiety and panic disorder. Retraining attention away from the body and panic-like thoughts.
Module 8. *Changing maladaptive conscious cognitions*	The importance of catastrophic thoughts and attitudes. ICAR technique for cognitive restructuring:
	Identify catastrophic cognitions
	Challenge each dysfunctional cognition
	Find the Alternative cognition
	Repeat the procedure
Module 9. *Changing maladaptive unconscious cognitions*	Cognitive restructuring techniques for unconscious beliefs. Identify personal values and live by them.
Module 10. *Avoidance reduction through interoceptive exposure*	Explain the mechanism of avoidance and prepare for exposure exercises. Reduce fear of bodily sensations through interoceptive exposure.
Module 11. *Avoidance reduction through exteroceptive exposure*	Reduce avoidance behaviors through gradual exposure to feared situations.
Module 13. *Positive emotions development*	Increase positive emotions in daily life through cognitive and behavioral exercises drawn from positive psychology practices.
Module 14. *Relapse prevention*	Resume the intervention strategies, set the correct expectancies and make a plan to continue improvement and deal with relapse situations.
Module 12. *You learn how to solve problems (optional)*	Learn to organize problems according to importance and urgency. Implement problem-solving technique.
Module 15. *Reducing depressive symptoms associated with panic – behavioral activation (optional)*	Depression symptoms co-morbid to panic disorder. Behavioral activation techniques.
Module 16. *Reducing depressive symptoms associated with panic – cognitive restructuring (optional)*	ICAR technique for cognitive restructuring of dysfunctional cognitions that induce and maintain depression.

#### Guidance

Both treatment groups use the same intervention, but the first group also receives guidance from a licensed psychotherapist. The participants in this group have regular 15–30 min Skype sessions (the length depends on the complexity of the modules and the needs of each patient) with their psychotherapist. The first Skype session is introductory and takes place in the first week of the treatment, after the participant completed the module “Anxiety, from normality to pathology”. From then on, a new session is scheduled almost every week, but only after the participant has completed the corresponding modules from the recommended timetable. The sessions are scheduled via an encrypted asynchronous communication system available on the treatment platform. The participants can also use this system between two Skype sessions if they need further clarifications from their therapist.

In total, there are 10 Skype sessions programmed. During these sessions, held over secured Skype, the psychotherapist checks if the participant has completed and understood each module, answers questions, and helps the participant carry out the recommended exercises. The main purpose of each online session is to make sure that the patient has everything he needs in order to put the recommended exercises into practice regularly. That means, according to a behavioral model of change [84], the psychotherapist try to set the necessary antecedents by providing procedural knowledge (‘I know what I have to do and how to do it”), self-efficacy (“I believe I can do it”) and motivation (“I’m convinced that it is worth doing it”). The agenda for Skype sessions usually involves: review of the week and how the participant feels, checking the homework assigned in previous sessions, discussing the last module, demonstrating a certain exercise or practicing together with the participant and, at the end, planning homework that should be carried out until the next session.

For example, in the first and the second session the psychotherapist works with the patient to develop a case conceptualization in order to understand the origin of the problem and the mechanisms by which it is maintained. They then discuss the treatment plan so that the patient understands how the intervention will answer his/her needs. In the session following the third module, the psychotherapist briefly explains the hyperventilation process, describes and demonstrates the breathing control technique to the patient and then they practice it once together. In the end, the psychotherapist helps the patient find a time and place to practice the technique twice a day. After the interoceptive exposure module, the psychotherapist explains the rationale of the technique once more, encourages the patient and does a few exercises from the list together with the patient (e.g. breathing for 2 min through a straw, spin in a chair for 20–30 s, cover the head with a scarf or a jacket etc.).

The modules and the entire therapeutic environment of PAXonline are carefully designed in order to facilitate the psychotherapeutic process (e.g. personalization of intervention, organizing information and knowledge in learning objects and use of other effective chunking strategies, enhancing learnability by using multimedia content, use of three layers of information, providing immediate feedback to queries, teach-back exercises, providing a reward after each module completion, offering access to a personal portfolio and a virtual library etc.) [10, 85, 86]. The program also contains certain techniques aimed at improving adherence and compliance with the treatment, e.g. rewards after module completion (short movies that induce positive emotions), the “meditation of the day”, which is sent via e-mail every 5 days and contains a link to a short movie or animation that is meant to be inspirational and/ or motivational. The participants’ adherence is carefully monitored by measuring overall usage time, the modules and homework assignments completed, the time spent on each module and each module component.

#### Therapists

Three licensed psychotherapists with formal training in cognitive-behavioral therapy and a minimum of 3 years of clinical experience work with the participants allocated in the first treatment group. They have also participated in a 3 months blended course (3 face to face meetings plus online learning) prior to this study and were instructed in using the PAXonline platform and delivering CBT interventions online. The guidance will be delivered according to a standard treatment protocol, but it will also be personalized according to each participant’s need. During the study, the team meets once a month for discussions and the main researcher is available for contact at any time.

### Instruments

The selected instruments have already been well validated and are frequently used in CBT clinical trials for panic disorder in particular, as well as for other anxiety and mood disorders. Measures are taken at baseline (pre-treatment), 6 weeks after the intervention started (mid-treatment), directly after the intervention (post-treatment, 12 weeks after baseline), 1, 3, 6 and 12 months after the intervention ends. All self report measures are administered through the internet (secured links are used and all the data are encrypted) and the diagnostic interviews are conducted through secured Skype or by phone. The questionnaires, which were not yet validated for Romanian population, were previously translated in Romanian and underwent a rigorous back-translation process to ensure a good adaptation.

### Primary outcomes

The primary outcomes are the diagnostic status of the participants at post-assessment and symptoms of panic disorder as assessed with the PDSS-SR.

Panic Disorder Severity Scale – Self Report (PDSS-SR). PDSS is originally a face-to-face interview and was adapted to a self-report questionnaire by Houck et al. [87]. The scale contains seven items that measure the severity of seven dimensions of panic disorder and associated symptoms: 1) frequency of panic attacks; 2) distress during panic attacks; 3) anticipatory anxiety (worry about future panic attacks); 4) agoraphobic fear and avoidance; 5) interoceptive fear and avoidance (i.e., apprehension and avoidance of bodily sensations); 6) impairment of or interference with work functioning; and 7) impairment of or interference with social functioning. The PDSS-SR generates a total score ranging from 0 to 28, with a higher score indicating more severe panic symptoms. The questionnaire has good psychometric properties (Cronbach’s alpha = 0.92, test-retest reliability is 0.83) and is sensitive to changes following treatment [87]. A cut-off score of six may discriminate between the presence and absence of current DSM-IV panic disorder and a cut-off score of fourteen may discriminate between mild and severe panic disorder [82].

### Diagnostic interview

Panic Disorder is assessed using the Romanian adapted version of Psychiatric Diagnostic Screening Questionnaire (PDSQ) [88, 89], which comprises a self-report screening scale, followed by a semi-structured interview delivered by a clinician. The PDSQ scale has good psychometric properties. In the Romanian validation study the mean of the alpha coefficients was .85 and test–retest reliability was above .80 for nine subscales, with a mean test–retest of .85 [89]. A diagnosis of Panic Disorder is considered if the participant’s score reaches a value of at least 4 on the Panic scale. The same cutoff is applied for agoraphobia. Among Romanian psychiatric patients, these values offered the best balance between sensitivity and specificity: .90/.72 for panic disorder, and .95/.85 for agoraphobia [89].

Following the screening procedures, all eligible participants are interviewed by one of three experienced licensed clinical psychologists, based on the semi-structured interviews included in the PDSQ. This interview has been developed based on DSM-IV diagnosis criteria. In addition to Panic and Agoraphobia diagnoses, any other mental disorder that reached PDSQ screening cutoff point is assessed during the interview. In order to increase inter-raters agreement on assessment protocol, the assessors also participated in a two days training before study commencement. The diagnostic interviews are conducted before and after treatment by clinicians blind to the treatment group. At post-treatment diagnostic interview we are going to count missing data as equivalent to still meeting diagnostic criteria for panic disorder. This is the procedure that is recommended according to the intention-to-treat paradigm and was used in several other studies (e.g. Hedman et al. [42]).

### Secondary outcomes

Secondary outcome measures include quality of life, stress levels, depressive symptoms, catastrophic cognitions, body vigilance, and fear of bodily sensations.

#### Depression

The Patient Health Questionnaire (PHQ-9) is a widely used measure for depression. PHQ-9 contains nine items and covers nine criteria listed in the *Diagnostic and Statistical Manual of Mental Disorders,* 4th Edition (*DSM-IV*), in regard to depression [90], requiring respondents to rate the frequency of present difficulties during the past 2 weeks. Scores indicate presence and severity of depression, with a maximum score of 27 and a minimum score of 0. Scores of 5, 10, 15, and 20 indicate mild, moderate, moderately severe, and severe depression, respectively. The internal reliability of the English version of the PHQ-9 in a clinical population was in the range of 0.86–0.89 [90], which indicates good reliability. The test-retest reliability was also good, 0.84, and the correlation with interview results is very high, 0.84. Furthermore, sensitivity to change — an essential characteristic of measures used to monitor response to treatment — has been repeatedly established [90].

#### Functional impairment

Mundt, Marks, Shear, and Greist [91] developed the Work and Social Adjustment Scale (WSAS). The WSAS is a 5-item simple, reliable and valid measure and we chose it in order to evaluate the self-reported functional impairment produced by anxiety problems. The scale items encompass different domains of functioning and include the following: ability to work, home management, social leisure, private leisure, and ability to form and maintain close relationships. Each item is rated on a 9-point Likert-type scale, ranging from 0 (no impairment) to 8 (very severe impairment). The maximum total score is 40, with higher scores representing greater impairment. The WSAS has demonstrated good internal consistency (range between 0.70 and 0.94) and test-retest reliability (0.73) and is sensitive to patients’ perceptions of disorder severity [91].

#### Attention biases

Body Vigilance Scale (BVS) [92] . We chose this scale in order to detect any changes in attention vigilance toward body sensations, a problem targeted in our treatment program. The 4 items of BVS measure the degree of attentional focus on, and perceived sensitivity towards changes in bodily sensations, and the amount of time spent scanning for bodily sensations. Research suggests that the BVS has adequate internal consistency in clinical and nonclinical populations and can be used to assess changes in bodily attention during cognitive–behavioral treatment for panic disorder [92–94].

#### Fear of body sensations

Body Sensations Questionnaire (BSQ) [95]. BSQ was chosen in order to measure the “fear of anxiety” concept, an aspect that it is targeted in our intervention program. This is a 17 item self-report questionnaire and is used to assess the degree to which sensations associated with autonomic arousal (e.g., “feeling short of breath,” “heart palpitations”) are experienced as frightening. Each item is rated on a five-point scale ranging from 1 (not at all) to 5 (extremely). The BSQ has good internal consistency (*α* = 0.89) and high test–retest reliability (*r* = 0.79) [96]. BSQ is also sensitive to changes following treatment, so it is considered to be a good choice for measuring treatment outcomes [95].

#### Catastrophic cognitions relevant to panic attacks and agoraphobia

Panic Attack Cognition Questionnaire (PACQ). The PACQ [97] is a 25-item scale assessing the extent to which catastrophic cognitions dominated subject’s thoughts before, during, or after panic attacks. Participants are asked to rate each thought on a 4-point scale indicating their level of preoccupation with that thought from 0 = not at all to 3 = totally dominates my thoughts. The total PACQ score is the sum of the 25 item scores. This scale was composed from items generated from the DSM-III-R description of panic attacks, from ideation reported in the anxiety literature, and from interviews with patients. The fifth edition of DSM is currently in use, but analyzing the DSM versions, we could conclude that the panic disorder diagnostic criteria have not changed much from DSM-III-R to DSM-5 (APA, 2013). There is still the distinction between autonomic nervous system symptoms (11 symptoms) and catastrophic cognitions (fear of dying, fear of losing control or going crazy). In the DSM-5 panic disorder and agoraphobia are two separated diagnoses, but other than that there are no major changes, at least not concerning the catastrophic cognitions, which are assessed through PACQ. The PACQ has good internal consistency as calculated by Cronbach alpha (.88). It has also been shown to successfully differentiate between individuals with and without panic attacks [97].

Agoraphobic Cognitions Questionnaire (ACQ) [95]. The ACQ is a 14 item self-report questionnaire, which assesses the frequency of frightening or maladaptive thoughts about the consequences of panic and anxiety. Each item is rated on a five-point scale ranging from 1 (thought never occurs) to 5 (thought always occurs when I am nervous). The ACQ has shown good internal consistency (*α* = 0.82) and test–retest reliability (*r* = 0.79) [96]. ACQ was also proven to be sensitive to changes following treatment [95]. Both measures, PACQ and ACQ, were chosen because they measure catastrophic cognitions relevant to panic disorder and agoraphobia, a very important aspect that is directly targeted in our treatment.

#### Perceived stress

Perceived Stress Scale – 10 (PSS-10) is a short and easy to use questionnaire which proved acceptable psychometric properties and is recommended for the assessment of perceived stress, both in practice and research [98]. PSS-10 [99] measures the degree to which one perceives aspects of one’s life as uncontrollable, unpredictable, and overloading. Participants are asked to respond to each question on a 5-point Likert scale ranging from 0 (never) to 4 (very often), indicating how often they have felt or thought a certain way within the past month. Scores range from 0 to 40, with higher composite scores indicating greater perceived stress. The PSS-10 has demonstrated good internal consistency (0.89) and also good divergent and convergent validity [98, 100].

### Additional measures

#### Treatment credibility and patient expectancies

The Credibility/Expectancy Questionnaire (CEQ) of Devilly and Borkovec is used to measure expectancy for change and treatment credibility. It comprises six questions, four on “thinking” and two on “feeling” and they are rated on a Likert-type scale from 1 to 9. The scale has shown good internal consistency (expectancy, α = .79–.90; credibility, α = .81–.86) and test–retest reliability (expectancy, 0.82; credibility, 0 .75) [101]. CEQ is administered twice; after the first and the fourth Skype session for the guided participants, and at 2 and 6 weeks during intervention for the unguided participants.

#### Working alliance

Working Alliance Inventory-Short Form (WAI-S) [102, 103]. The WAI-S is a 12-item client self-report measure to assess the working relationship and therapeutic bond between client and therapist. This measure is commonly used in CBT treatment outcome studies’, including those in which treatment is delivered remotely. The 3 subscales are: agreement on goals for treatment, agreement on therapeutic tasks, and perception of therapeutic bond. Ratings are given on a 7 - point Likert scale, with higher scores reflecting a stronger working alliance. Busseri and Tyler [104] reported that the WAI - S has good internal reliability for the composite score (alpha = .91) and the subscales (alpha = .73 to .86). WAI-S is administered twice, after the first and the fourth Skype session, according to existing recommendations [105].

#### Personality traits (dependent personality and personal autonomy)

OMNI-IV [106]. Derived from the 375-item OMNI Personality Inventory, the 210-item OMNI-IV measures abnormal personality traits and assesses personality disorders. It is composed of 10 Personality Disorder scales based on the DSM-IV Axis II personality disorder criteria. For our study we use the Dependent Personality Subscale, which has 15 items. Previous studies showed good internal consistency, α = .80–.82 and also test-retest reliability, 0.80–0.84 [106]. In the Romanian sample used to validate the scale internal consistency varied between 0.79 and 0.88 and test-retest reliability was 0.67 [107].

Personal Autonomy Questionnaire (PAQ) [108]. The PAQ is a 36-item questionnaire designed to measure four dimensions of personal autonomy: cognitive autonomy, behavioral autonomy, emotional autonomy and value autonomy. Ratings are given on a 5-point Likert scale, with higher scores reflecting a stronger personal autonomy (reverse score items: 1, 4, 12, 13, 14, 15, 19, 29, 34, 35 and 36). PAQ has good internal consistency, α = .80 and also test-retest reliability, 0.86. [108].

#### Perceived social support

The SS-5 is an abbreviated version of the Medical Outcomes Study Social Support Scale (MOS-SSS) [109]. This is a 5 item short and reliable measure for assessing perceived social support, a putative predictor or moderator of treatment outcome. Each item is scored on a 5-point Likert scale and the items are summed for a total score ranging from 5–25. The SS-5 proved to have adequate test-retest reliability (0.92) and internal consistency (α = .88). Equivalence between paper and web-based administration has also been demonstrated [110].

#### Usability

The System Usability Scale (SUS) is one of the oldest questionnaires used to evaluate usability of a system and was designed in 1986 by Broke. The SUS consists of 10 items scored on a 5-point Likert scale (1-5), with odd-numbered items worded positively and even-numbered items worded negatively. The scale has been used in hundreds of studies and the psychometric qualities are adequate (α = .88). For many years the scale was considered unidimensional, but recent studies have shown that it measures not just usability, but also learnability of a new system [111].

#### Sociodemographic questionnaire

To evaluate the sociodemographic characteristics of the participants, we used a 7 questions survey referring to: environment (urban or rural), ethnicity, marital status, education, occupation, income levels and computer skills (1 to 10).

#### Psychoeducation

Psycho-Education Questionnaire (PEQ)*.* In order to evaluate the information participants have about panic disorder and its treatment, we used a brief knowledge test that consisted of an 8-item questionnaire. Seven of the questions used are multiple choice questions and the last one is a self-evaluation question concerning the level of knowledge about PD and its treatment (from 1 – “I don’t know anything”, to 10,” I know almost everything”).

#### Adherence to treatment and homework compliance

Adherence to treatment and completion rates will be assessed. Number of modules completed, time spent in total, time spent on each module, and usage of the support will be analyzed to identify potential mediating effects.

#### Frequency of use of anxiety reduction strategies

In order to assess how frequent the anxiety reduction techniques were used we have developed a 10 items questionnaire. Eight of these questions are scored on a 5-point Likert scale (0-never and 4-daily or almost daily) and the last two are open ended questions: “Which techniques helped you the most?” and “Which are the techniques that you plan on using after the end of the intervention?”

#### Patient satisfaction

The Patient Feedback Questionnaire (PFQ). We have developed a 14-items questionnaire to evaluate the patients’ satisfaction with the platform and some other aspects (the quality and utility of different components of the platform). Other items refer to giving improvement suggestions, what they like most, if they would use it again or recommend it to a friend with anxiety problems.

#### Drop-out reasons

In order to find the reasons or motives participants had for dropping out of the study, we have developed an 18-item questionnaire. Each item is considered to be a possible motive (e.g. lack of motivation, lack of time, different expectations, exacerbation of symptoms, interface is too complicated, the treatment pace is too slow or too fast, finding another treatment, getting better, important life events etc.).

### Randomization

Participants who return the informed consent and meet all the required criteria are randomly allocated to one of the three conditions (1:1:1 allocation ratio). The randomization process is done by a software that was developed to implement a minimization algorithm [112], that assures a balanced randomization between groups with respect to certain predefined prognostic (stratification) factors. In this study, two stratification factors have been considered: SEVERITY (3 levels according to PDSS-SR screening scores; level 1: 6 to 13 points; level 2: 14 to 20 points; level 3: 21 to 28 point) and CHRONICITY (2 levels; more or less than 6 months since the beginning of PD) of panic disorder. The minimization method has been shown to outperform simple randomization in achieving balanced groups [113] and its use in clinical trials has been previously recommended as a better option than other randomization methods (e.g. [113–115]). Before each allocation, the algorithm computes an imbalance score for the three available treatments, taking stratification factors levels into account. The treatment with the lowest imbalance score is then given preference when assigning treatments, but the allocation probability varies for each patient, depending on the actual level of imbalance [116]. This method is preferred because it avoids the deterministic allocation of pure minimization [117]. The allocation is done by an independent researcher and is concealed, i.e. patients and researchers have no knowledge and no control over the allocation of participants when they randomize a participant with the computer program.

### Blinding

Taking the characteristics of our research into consideration, it is impossible to keep patients or psychotherapists blind to the study procedures and intervention. All participants are provided with detailed information about the aims and the methodology of the study. They can request more information about the study and they have the right to terminate participation at any time. The personnel in charge with the clinical interviews, on the other hand, will be blind to the treatment group allocation. We specifically instruct the participants not to mention group allocation at the post-treatment interview, but also test whether blinding was successful. The clinicians who conduct the diagnostic interviews are required to describe in their report if the participants disclosed their study group, directly or indirectly.

### Sample size

The effect sizes reported between guided and unguided interventions are quite heterogeneous with small to medium Cohen’s d size effects between 0.27 [64] and .42 [14]. Taking into consideration that, in most part, the guidance in previous studies was in fact low-intensity guidance, and that more support from a therapist yields larger effects [21, 62, 63], we expect an effect size of 0.5 (Cohen’s d) between guided and unguided treatment for PD. Based on this effect size, a power of 0.80, and an alpha level of 0.05, we would need 51 subjects in each condition. This recommended sample size has been increased to accommodate an attrition rate of 20 %. Thus, the study aims to achieve a sample of 192 participants.

### Statistical analysis

All statistical analyses will be performed using the IBM SPSS Statistics version 20. The main analyses will be conducted on the intention-to-treat samples (all randomised participants, regardless of protocol adherence). Following current standards [27, 28], a linear mixed effects models approach with full information maximum likelihood estimation will be used. This approach has been recommended since it uses all available data and can handle missing data appropriately [118, 119]. The approach is based on the assumption that data are missing at random, and does not assume that missing data remain stable, as in the last-observation-carried-forward (LOCF) approach [119, 120]. Significance testing of dichotomous data such as diagnostic status will be conducted with chi-square tests. Calculations of within- and between-groups effect sizes (Cohen’s d) will be based on the pooled standard deviations. Regression analyses will be used to identify predictors of treatment outcome. Moderation and mediation analyses will be conducted with multiple regression models, using the PROCESS macro for SPSS [121], a computational technique that can compute both simple and complex moderation and mediation models. Effect sizes for significant effects will be indicated by Cohen’s f.

Consistent with CONSORT [77] and SPIRIT [78] recommendations we mention our intention to perform secondary analyses for minimal treatment users, defined as participants that complete at least 5 of the recommended modules, have at least 3 of the available guidance sessions (this is available only for the Skype-guided group) and use the intervention program for at least 80 min. This definition is based on the specific of our intervention program and the criteria used in other studies [27, 119].

## Discussion

The prevalence of mental health problems is very high and bears considerable suffering and burden worldwide. However, only a small percentage of the affected individuals reach out and receive treatment for their problems. There are many barriers at different levels, yet a viable solution to increase access to quality treatment resides in the possibilities provided by internet-based cognitive behavioral treatments. Although several ICBT programs have been tested with strong positive results and even integrated into regular health care, there are countries where validated internet-based interventions are yet to be developed. The present trial aims both to validate the overall efficacy of the first Romanian ICBT program for panic disorder, and to investigate aspects that are novel to ICBT, such as brief therapist guidance sessions via secured Skype.

There are still divergent results regarding the effect of therapist guidance in ICBT [18, 31, 56–58], and the efficacy of Skype-based (or equivalent) therapy sessions in ICBT have not been previously investigated. Thus, a central focus of our trial is to test the additive effect of therapeutic guidance via secured Skype in self-help ICBT programs. Specifically, the current study design allows us to assess the effect of the same self-help intervention modules in two treatment conditions: therapist guided via Skype vs. unguided self-directed intervention.

There is a general consensus that internet-based therapy can be efficient for many psychiatric conditions, but not all the patients benefit from it [44]. Thus, there is an increasing interest to describe the characteristics of the patients who would benefit the most from this type of interventions. Taking into consideration this issue, additional exploratory analyses of potential predictors, moderators or mediators will be made. Patients’ treatment expectations, personality traits (e.g. personal autonomy), and usability of the online treatment program could prove to be key factors of treatment adherence and outcome, especially in the unguided self-help treatment group.

Several possible limitations should be noted. Firstly, the study sample size is large enough to reach our main research objectives, but will not permit us to perform detailed subgroup analyses. However, we expect to identify candidate predictors of treatment outcome that could be tested directly in future studies with larger samples. Secondly, the control group, for ethical reasons, will receive treatment in the second stage of the trial, thus the control group will not be available at follow-up. Furthermore, most of the recruitment will be made from the community, through advertisements in the media and on the study website, which could lead to a self-selected sample. Regarding the latter limitation, several findings from recent studies suggest that sampling bias may not affect the generalizability of internet-based trials. For example, Titov et al. [122] revealed that patients undergoing web-based treatments resemble national samples and present disorders as severe as those undergoing traditional psychotherapy. Another example is a recent study by Donkin et al. [123] on sampling bias in an internet-based clinical trial for depression. The authors revealed that out of 35 potentially biasing factors, only 4 were associated with consenting likelihood, accounting for limited variance in explaining participation, and none were associated with the primary outcomes. Future research will be required, nonetheless, to investigate whether the results from the PAXonline trial can be generalized to samples in regular mental health care settings.

In Romania, the need for easily accessible treatment programs for mental disorders is very high. 76.4 % of the individuals with mental health problems do not have access to any form of treatment, and only approximately 11.5 % receive psychological or psychiatric treatment [12]. Internet-based treatments can offer a viable alternative solution to the current situation, considering that internet access has increased rapidly over the past years in Romania (in 2004 only 6 % households had access to the internet, and presently the number has increased to 61 % [124]). However, although ICBT advantages are easily recognized, both patients and psychologists in Romania have a reserved attitude towards using internet-based psychological intervention [125]. By developing rigorous clinical trials on the efficiency of Romanian ICBT programs, we can increase the uptake of these solutions, and thus have a significant positive impact on the quality of life, both at an individual and national level.

## Trial status

Trial start date: May 22, 2014.

Currently recruiting (N_current_ = 99 as of December 15, 2015).
